# Crystal structure of tetra­kis­(1,1,1,5,5,5-hexa­fluoro­acetyl­acetonato)hafnium(IV)

**DOI:** 10.1107/S2056989018010514

**Published:** 2018-07-31

**Authors:** W. Franklin Schwandt, Toby J. Woods, Gregory S. Girolami

**Affiliations:** a School of Chemical Sciences, University of Illinois at Urbana-Champaign, 600 South Mathews Avenue, Urbana, IL 61801, USA; b George L. Clark X-Ray Facility and 3M Materials Laboratory, University of Illinois at Urbana-Champaign, 505 South Mathews Avenue, Urbana, IL 61801, USA

**Keywords:** crystal structure, hafnium, hexa­fluoro­acetyl­acetonate

## Abstract

The crystal structure of the title compound, [Hf(C_5_HF_6_O_2_)_4_], has been determined. The asymmetric unit contains two Hf(hfac)_4_ mol­ecules (hfac = 1,1,1,5,5,5-hexa­fluoro­acetyl­acetonate); both are located on general positions and have identical structures apart from the disorder involving three CF_3_ groups in one of the two mol­ecules. The mol­ecules of Hf(hfac)_4_ are arranged in layers that are parallel to the *ab* plane, and the coordination geometry of each hafnium(IV) center is a distorted square anti­prism. An inter­esting aspect of the structure is that the hfac ligands are arranged so that the Hf(hfac)_4_ mol­ecules have idealized 2 point symmetry, in which two of the hfac groups bridge between the two squares. Although all other *M*(β-diketonate)_4_ compounds of Hf (and Zr) also have square-anti­prismatic geometries; in almost all of them the ligands are arranged so that the mol­ecules have 222 point symmetry (in which none of the hfac ligands bridges between the two squares). The factors that favor one structure over another are not clear.

## Abstract 2

The crystal structure of the square-anti­prismatic complex tetra­kis­(1,1,1,5,5,5-hexa­fluoro­acetyl­acetonato)hafnium(IV) is reported.

## Chemical context   

The mol­ecule tetra­kis­(1,1,1,5,5,5-hexa­fluoro­acetyl­acetonato)hafnium(IV), Hf(hfac)_4_, has a relatively high vapor pressure for a hafnium compound, and in part for this reason it has been identified as a potential chemical vapor deposition (CVD) precursor for thin films of hafnium dioxide (Balog *et al.*, 1977 ▸; Morozova *et al.*, 2008 ▸; Wilk *et al.*, 2001 ▸; Zherikova & Morozova, 2012 ▸; Zherikova *et al.*, 2008 ▸).
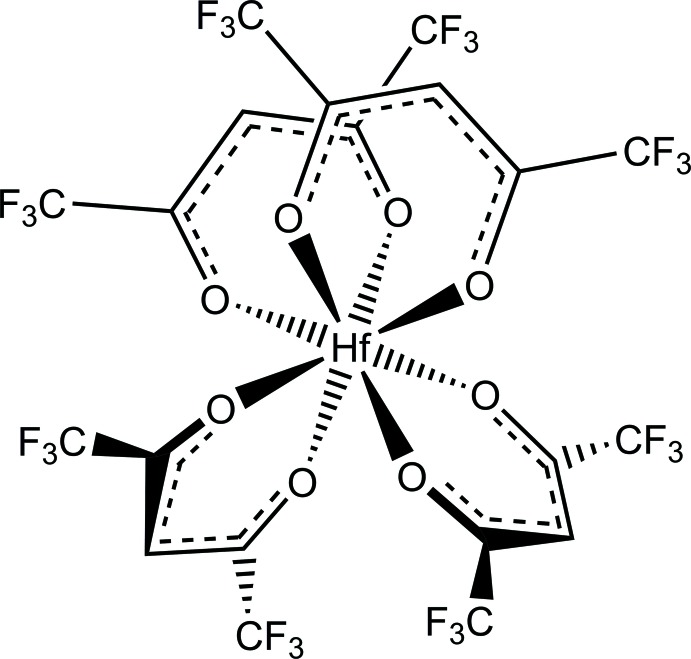


Thin films of HfO_2_ are widely used as the gate oxide in integrated circuits because of its high dielectric constant. Although most CVD precursors for HfO_2_, such as the di­alkyl­amide Hf(NMe_2_)_4_, have mol­ecular weights less than 500, it has recently been discovered that higher mol­ecular weight precursors can enable superconformal growth in high aspect ratio features (*i.e*., faster growth deeper in the feature), which is an important goal in the microelectronics industry (Wang *et al.*, 2014 ▸). The mol­ecular weight of Hf(hfac)_4_ is quite high (1006.8), but it nevertheless is highly volatile owing to the fluorine substit­uents, which reduce the strength of inter­molecular inter­actions (Jones *et al.*, 2009 ▸). Here we report on the crystal structure of Hf(hfac)_4_.

## Structural commentary   

**Figure 1:**
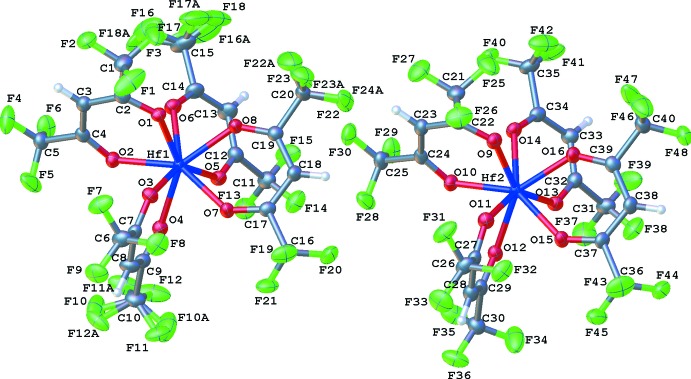
The asymmetric unit of Hf(hfac)_4_ with displacement ellipsoids drawn at the 50% probability level. F10*A*–F12*A*, F16*A*–F18*A* and F22*A*–F24*A* are the minor components of the F atoms of the disordered CF_3_ groups in the Hf1 molecule. Color coding: C – grey, O – red, H – white, F– green, Hf – blue.

**Figure 2:**
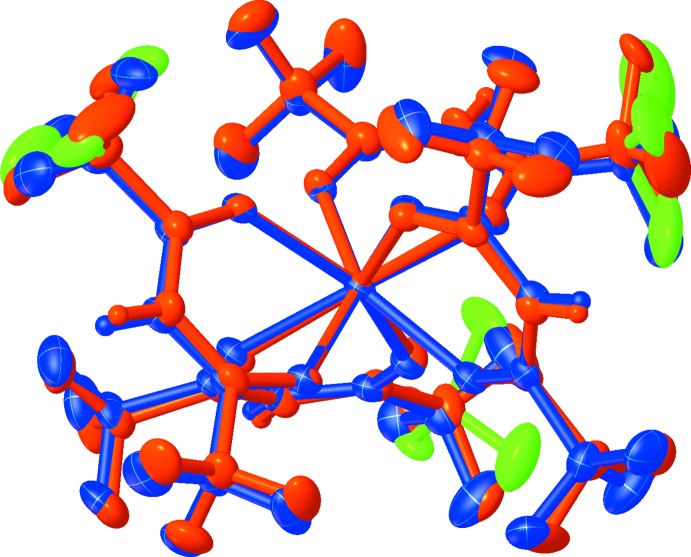
Overlay of the crystallographically inequivalent Hf(hfac)_4_ mol­ecules with mol­ecule 1 in orange, mol­ecule 2 in blue, and the disordered CF_3_ groups in green; r.m.s. deviation = 0.004 Å.

There are two crystallographically inequivalent Hf(hfac)_4_ mol­ecules in the asymmetric unit (Fig. 1 ▸). The two mol­ecules are structurally identical (r.m.s. deviation = 0.004 Å), except that three of the CF_3_ substituents in the Hf1 mol­ecule are disordered over two sites (Fig. 2 ▸). Each hafnium atom is bound to four bidentate hfac ligands; the eight oxygen atoms define a square anti­prism in which two of the hfac ligands bridge between the squares, giving an idealized point symmetry of 2. The Hf—O bond lengths range from 2.134 (2) to 2.210 (3) Å, whereas the C—O bond lengths range from 1.247 (5) to 1.275 (5) Å. All of the distances are as expected except that the C—F bond distances vary over a larger range than usual owing to the disorder.

## Supra­molecular features   

**Figure 3:**
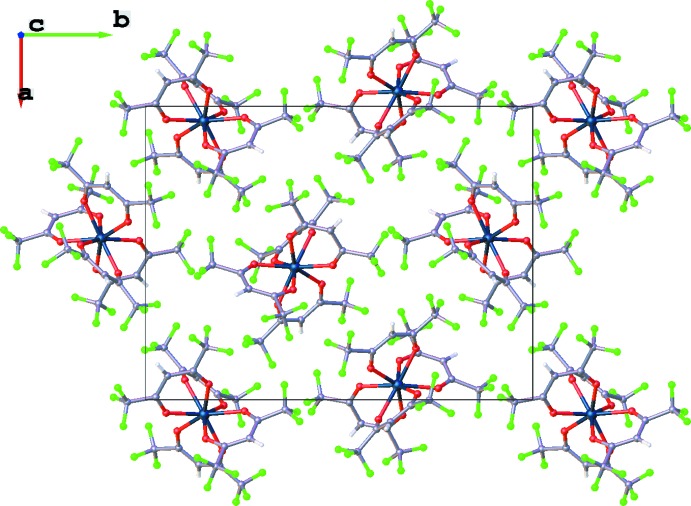
A single layer of Hf(hfac)_4_ mol­ecules as viewed along the *c* axis.

The mol­ecules are well separated and the shortest Hf⋯Hf distance is 8.3610 (1) Å. The mol­ecules form layers parallel to the *ab* plane (Figs. 3 ▸ and 4 ▸). Of the inter­molecular C—H⋯F contacts (ignoring the minor site F atoms; Table 1 ▸), only three are comparable to the 2.60 Å sum of the van der Waals radii (1.2 Å for H, and 1.40 Å for F attached to a primary alkyl (Bondi, 1964 ▸): H8⋯F5 (2.50 Å), H13⋯F35 (2.65 Å), and H3⋯F24 (2.68 Å). Only one inter­molecular F⋯F inter­action (again ignoring the minor site F atoms) is shorter than 2.8 Å, the sum of van der Waals radii for two F atoms attached to a primary alkyl, that involving F2⋯F22 (2.76 Å). All these inter­molecular contacts must be weak, given that the compound sublimes in moderate vacuum only slightly above room temperature.

## Database survey   

A search of the Cambridge Structural Database (CSD) returned 21 structures of the form Hf(*R*COCHCO*R*′)_4_ and 21 of the form Zr(*R*COCHCO*R*′)_4_ (Groom *et al.*, 2016 ▸). The *R* and *R*′ groups, which could either be the same or different, included Me, CF_3_, ^*i*^Pr, CH_2_^*t*^Bu, ^*t*^Bu, thio­furanyl, CMe_2_(OMe), OSiMe_3_, OMe, OEt, O^*t*^Bu, Ph, and CH_2_COO^*t*^Bu. In all cases, the eight oxygen atoms describe a square anti­prism about the metal center. This is the geometry expected for *M*(bidentate)_4_ mol­ecules in which the bidentate ligand has a large bite angle (Kepert, 1982 ▸).

**Figure 5:**
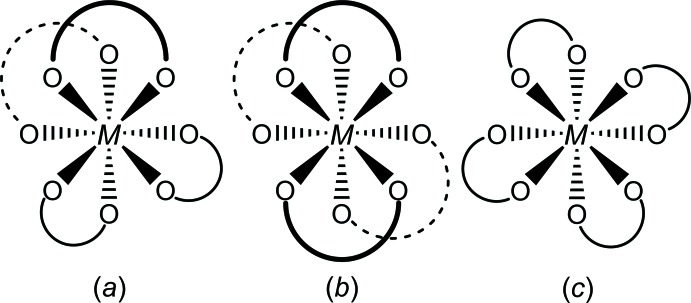
The three different isomers of square anti­prismatic tetra­kis­(bidentate) metal complexes. Point symmetries: (*a*) 2, (*b*) 222, (*c*) 422.

Inter­estingly, of the 42 structures in the CSD, three different arrangements of the ligands have been seen, corres­ponding to the three idealized mol­ecular point symmetries possible for a square-anti­prismatic coordination geometry with four bidentate ligands (Fig. 5 ▸) (Marchi *et al.*, 1943 ▸; Hoard & Silverton, 1963 ▸; Muetterties & Wright, 1967 ▸). The majority of them describe mol­ecules with idealized 222 symmetry (in which none of the ligands bridge between the two squares), two describe mol­ecules with idealized point symmetries of 2 (in which two of the ligands bridge between the two squares), and one describes a mol­ecule with idealized 422 symmetry (in which all four ligands bridge between the two squares; in all cases, these point symmetries describe the arrangement of the ligands, and neglect differences between the *R* and *R*′ groups, if any).

The current mol­ecule Hf(hfac)_4_ adds to the small number of group 4 *M*(*R*COCHCO*R*′)_4_ complexes that adopt the structure with an idealized point symmetry of 2; inter­estingly, one of the others is the zirconium analog Zr(hfac)_4_ (Calderazzo *et al.*, 1998 ▸). There is no obvious reason why Hf(hfac)_4_ and Zr(hfac)_4_ adopt this geometry rather than one of the other two. Irrespective of the structure adopted, the Hf—O and Zr—O bond distances in all Zr and Hf β-diketonates are all near 2.2 Å.

The NMR data for Hf(hfac)_4_ show that all four C—H groups and all eight CF_3_ groups are chemically equivalent on the NMR time scale at room temperature, so that there must be a dynamic process that inter­converts the different hfac environments.

## Synthesis and crystallization   

To a mixture of sodium 1,1,1,5,5,5-hexa­fluoro­acetyl­acetonate (Harada & Girolami, 2007 ▸) (1.46 g, 6.36 mmol) and hafnium tetra­chloride (0.51 g, 1.59 mmol) at 195 K was added diethyl ether (10 mL). The mixture was warmed to room temperature and allowed to stir overnight. The solution was filtered, and the filtrate was taken to dryness under vacuum. The colorless product Hf(hfac)_4_ was sublimed out of the brown residue at 15 mTorr and 303 K onto a water-cooled cold finger. Yield: 0.44 g (28%). ^1^H NMR (400 MHz, C_6_D_6_): δ 6.12 (*s*). ^19^F NMR (400 MHz, C_6_D_6_): δ −77.01 (*s*). The NMR spectra are similar to those previously reported for this compound in CCl_4_ (^1^H NMR: δ 6.54; ^19^F NMR: δ −74.7); note that this previous work used the opposite chemical shift sign convention and a different ^19^F NMR shift reference (Chattoraj *et al.*, 1968 ▸).

X-ray quality crystals were grown by allowing Hf(hfac)_4_ (0.1 g) to sublime inside an evacuated 50 mL Schlenk tube placed on top of a warm oven. After 12 h, crystals had formed on the cooler parts of the tube.

## Refinement   

Crystal data, data collection and structure refinement details are summarized in Table 2 ▸. H-atom positions were positioned geometrically and refined as riding: C—H = 0.95 Å with *U*_iso_(H) = 1.2*U*_eq_(C). The F10–F12, F16–F18, and F22–F24 atoms are disordered over two sites; their occupancies refine to 0.644 (18):0.356 (18), 0.507 (6):0.493 (6) and 0.61 (2):0.39 (2), respectively. Within each disordered CF_3_ group, the C—F distances were restrained to 1.35±0.01 Å, and the F—C—F and C—C—F bond angles were limited to near-tetra­hedral values by restraining the F⋯F and β-C⋯F distances to 2.15 (1) and 2.3 (5) Å, respectively. The displacement parameters for all F atoms were restrained to be approximately isotropic (ISOR 0.005). The (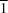
11), (021), (011), (110), (113), (122), (111), (220), and (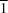
21) reflections were obscured by the beam stop and were omitted from the final refinement. The largest electron density peak in the difference map (4.36 e Å^−3^) is located 0.85 Å from Hf2 and is certainly a Fourier truncation ripple.

## Supplementary Material

Crystal structure: contains datablock(s) I. DOI: 10.1107/S2056989018010514/sj5560sup1.cif

Structure factors: contains datablock(s) I. DOI: 10.1107/S2056989018010514/sj5560Isup2.hkl

CCDC reference: 1857253

Additional supporting information:  crystallographic information; 3D view; checkCIF report

## Figures and Tables

**Figure 4:**
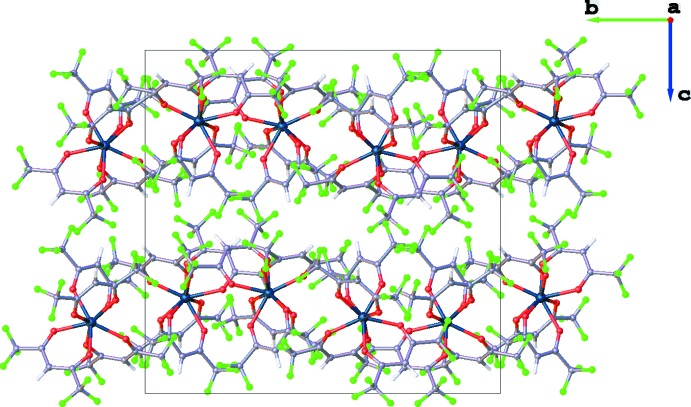
The layered structure of Hf(hfac)_4_ as viewed along the *a* axis.

**Table 1:** Hydrogen-bond geometry (Å, °)

*D*—H⋯*A*	*D*—H	H⋯*A*	*D*⋯*A*	*D*—H⋯*A*
C3—H3⋯F23*A* ^i^	0.95	2.57	3.374 (10)	143
C3—H3⋯F24^i^	0.95	2.68	3.375 (8)	130
C8—H8⋯F5^ii^	0.95	2.50	3.427 (5)	166
C33—H33⋯F11*A* ^iii^	0.95	2.49	3.292 (9)	142
C13—H13⋯F35^iii^	0.95	2.65	3.528 (5)	154

Symmetry codes: (i) 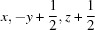; (ii) 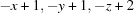; (iii) 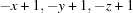.

**Table 2:** Experimental details

Crystal data	
Chemical formula	[Hf(C_5_HF_6_O_2_)_4_]
*M*_r_	1006.72
Crystal system, space group	Monoclinic, *P*2_1_/*c*
Temperature (K)	100
*a*, *b*, *c* (Å)	15.3042 (3), 20.0723 (4), 19.4935 (4)
β (°)	96.158 (1)
*V* (Å^3^)	5953.7 (2)
*Z*	8
Radiation type	Mo *K*α
μ (mm^−1^)	3.70
Crystal size (mm)	0.23 × 0.22 × 0.19

Data collection	
Diffractometer	Bruker APEXII CCD
Absorption correction	Multi-scan (*SADABS*; Krause *et al.*, 2015 ▸)
*T*_min_, *T*_max_	0.661, 0.746
No. of measured, independent and observed [*I* > 2σ(*I*)] reflections	211180, 14806, 12738
*R*_int_	0.044
(sin θ/λ)_max_ (Å^−1^)	0.668

Refinement	
*R*[*F* ^2^ > 2σ(*F* ^2^)], *wR*(*F* ^2^), *S*	0.031, 0.074, 1.10
No. of reflections	14806
No. of parameters	1039
No. of restraints	369
H-atom treatment	H-atom parameters constrained
Δρ_max_, Δρ_min_ (e Å^−3^)	4.36, −1.89

Computer programs: *APEX2* and *SAINT* (Bruker, 2014 ▸), *SHELXT2014* (Sheldrick, 2015*b* ▸), *SHELXL2014* (Sheldrick, 2015*a* ▸) and *OLEX2* (Dolomanov *et al.*, 2009 ▸).

## supplementary crystallographic information

### Crystal data

**Table:**

[Hf(C_5_HF_6_O_2_)_4_]	*F*(000) = 3808
*M**_r_* = 1006.72	*D*_x_ = 2.246 Mg m^−^^3^
Monoclinic, *P*2_1_/*c*	Mo *K*α radiation, λ = 0.71073 Å
*a* = 15.3042 (3) Å	Cell parameters from 58639 reflections
*b* = 20.0723 (4) Å	θ = 2.3–28.2°
*c* = 19.4935 (4) Å	µ = 3.70 mm^−^^1^
β = 96.158 (1)°	*T* = 100 K
*V* = 5953.7 (2) Å^3^	Prism, colourless
*Z* = 8	0.23 × 0.22 × 0.19 mm

### Data collection

**Table:**

Bruker APEXII CCD diffractometer	12738 reflections with *I* > 2σ(*I*)
φ and ω scans	*R*_int_ = 0.044
Absorption correction: multi-scan (SADABS; Krause *et al.*, 2015)	θ_max_ = 28.3°, θ_min_ = 1.3°
*T*_min_ = 0.661, *T*_max_ = 0.746	*h* = −20→20
211180 measured reflections	*k* = −26→26
14806 independent reflections	*l* = −26→26

### Refinement

**Table:**

Refinement on *F*^2^	369 restraints
Least-squares matrix: full	Hydrogen site location: inferred from neighbouring sites
*R*[*F*^2^ > 2σ(*F*^2^)] = 0.031	H-atom parameters constrained
*wR*(*F*^2^) = 0.074	*w* = 1/[σ^2^(*F*_o_^2^) + (0.0259*P*)^2^ + 18.914*P*] where *P* = (*F*_o_^2^ + 2*F*_c_^2^)/3
*S* = 1.10	(Δ/σ)_max_ = 0.003
14806 reflections	Δρ_max_ = 4.36 e Å^−^^3^
1039 parameters	Δρ_min_ = −1.89 e Å^−^^3^

### Special details

**Table:**

Geometry. All esds (except the esd in the dihedral angle between two l.s. planes) are estimated using the full covariance matrix. The cell esds are taken into account individually in the estimation of esds in distances, angles and torsion angles; correlations between esds in cell parameters are only used when they are defined by crystal symmetry. An approximate (isotropic) treatment of cell esds is used for estimating esds involving l.s. planes.

### Fractional atomic coordinates and isotropic or equivalent isotropic displacement parameters (Å^2^)

**Table:**

	*x*	*y*	*z*	*U*_iso_*/*U*_eq_	Occ. (<1)
Hf1	0.54873 (2)	0.38630 (2)	0.77866 (2)	0.01927 (4)	
O1	0.53844 (16)	0.28122 (12)	0.80247 (12)	0.0205 (5)	
O2	0.62501 (17)	0.38168 (13)	0.87740 (13)	0.0241 (5)	
C1	0.5399 (3)	0.1738 (2)	0.8474 (2)	0.0350 (9)	
F1	0.45392 (19)	0.16898 (15)	0.8499 (2)	0.0679 (10)	
F2	0.57856 (17)	0.13531 (12)	0.89695 (13)	0.0370 (6)	
F3	0.5599 (3)	0.14822 (14)	0.78807 (15)	0.0677 (10)	
C2	0.5686 (2)	0.24662 (19)	0.85349 (19)	0.0236 (7)	
C3	0.6221 (2)	0.2677 (2)	0.91177 (19)	0.0250 (7)	
H3	0.6416	0.2367	0.9470	0.030*	
C4	0.6470 (2)	0.33396 (19)	0.91818 (18)	0.0228 (7)	
C5	0.7101 (3)	0.3559 (2)	0.9803 (2)	0.0282 (8)	
F4	0.72420 (19)	0.30906 (16)	1.02815 (13)	0.0514 (7)	
F5	0.6798 (2)	0.40941 (17)	1.00909 (15)	0.0596 (9)	
F6	0.78750 (16)	0.37207 (15)	0.96098 (13)	0.0419 (6)	
O3	0.44467 (16)	0.37390 (13)	0.84384 (13)	0.0233 (5)	
O4	0.54190 (16)	0.48451 (13)	0.82534 (13)	0.0231 (5)	
C6	0.3275 (3)	0.3795 (2)	0.9110 (2)	0.0315 (9)	
F7	0.34891 (17)	0.31919 (13)	0.93486 (15)	0.0447 (7)	
F8	0.26091 (15)	0.37342 (14)	0.86177 (14)	0.0428 (6)	
F9	0.29883 (17)	0.41418 (14)	0.96194 (14)	0.0441 (6)	
C7	0.4053 (2)	0.41242 (19)	0.88110 (18)	0.0229 (7)	
C8	0.4239 (3)	0.4783 (2)	0.8948 (2)	0.0301 (8)	
H8	0.3900	0.5027	0.9242	0.036*	
C9	0.4925 (2)	0.50954 (19)	0.86582 (19)	0.0244 (7)	
C10	0.5104 (3)	0.5827 (2)	0.8848 (2)	0.0337 (9)	
F10	0.5280 (9)	0.5913 (5)	0.9485 (4)	0.075 (3)	0.644 (18)
F11	0.4377 (3)	0.6185 (3)	0.8640 (4)	0.0410 (16)	0.644 (18)
F12	0.5689 (5)	0.6093 (3)	0.8483 (6)	0.061 (2)	0.644 (18)
F10A	0.4662 (13)	0.6251 (7)	0.8458 (7)	0.082 (5)	0.356 (18)
F11A	0.5975 (6)	0.5965 (7)	0.8883 (10)	0.066 (4)	0.356 (18)
F12A	0.4944 (9)	0.5948 (8)	0.9513 (5)	0.042 (3)	0.356 (18)
O5	0.60640 (16)	0.45575 (13)	0.71347 (13)	0.0240 (5)	
O6	0.67729 (16)	0.34269 (13)	0.76348 (14)	0.0258 (5)	
C11	0.6729 (3)	0.5152 (2)	0.6296 (2)	0.0324 (9)	
F13	0.69778 (19)	0.56731 (13)	0.66855 (14)	0.0458 (7)	
F14	0.59588 (18)	0.52931 (14)	0.59612 (14)	0.0461 (7)	
F15	0.7301 (2)	0.50791 (15)	0.58351 (16)	0.0560 (8)	
C12	0.6679 (2)	0.4527 (2)	0.67444 (19)	0.0270 (8)	
C13	0.7281 (3)	0.4021 (2)	0.6715 (2)	0.0347 (9)	
H13	0.7680	0.4024	0.6374	0.042*	
C14	0.7295 (2)	0.3506 (2)	0.7193 (2)	0.0297 (8)	
C15	0.8009 (3)	0.2969 (3)	0.7202 (3)	0.0502 (13)	
F16	0.8107 (7)	0.2602 (5)	0.7724 (5)	0.076 (4)	0.507 (6)
F17	0.8811 (3)	0.3284 (3)	0.7178 (3)	0.0359 (14)	0.507 (6)
F18	0.7919 (5)	0.2634 (5)	0.6628 (5)	0.075 (3)	0.507 (6)
F16A	0.8637 (7)	0.3108 (6)	0.6835 (7)	0.134 (5)	0.493 (6)
F17A	0.7623 (6)	0.2390 (4)	0.6979 (6)	0.096 (4)	0.493 (6)
F18A	0.8322 (7)	0.2835 (5)	0.7855 (4)	0.082 (4)	0.507 (6)
O7	0.43069 (16)	0.43040 (13)	0.72452 (12)	0.0234 (5)	
O8	0.52769 (17)	0.33355 (13)	0.68213 (13)	0.0244 (5)	
C16	0.3184 (3)	0.4779 (2)	0.6483 (2)	0.0299 (8)	
F19	0.24631 (16)	0.44484 (13)	0.66136 (16)	0.0458 (7)	
F20	0.3066 (2)	0.49750 (15)	0.58295 (14)	0.0522 (8)	
F21	0.32427 (18)	0.53092 (12)	0.68787 (14)	0.0432 (6)	
C17	0.3988 (2)	0.43221 (19)	0.66260 (19)	0.0242 (7)	
C18	0.4258 (2)	0.3952 (2)	0.6084 (2)	0.0263 (8)	
H18	0.4022	0.4036	0.5621	0.032*	
C19	0.4879 (2)	0.34600 (19)	0.62377 (19)	0.0242 (7)	
C20	0.5124 (3)	0.3000 (2)	0.5672 (2)	0.0333 (9)	
F22	0.4891 (8)	0.3242 (6)	0.5060 (5)	0.044 (2)	0.61 (2)
F23	0.4621 (7)	0.2422 (3)	0.5695 (4)	0.050 (2)	0.61 (2)
F24	0.5912 (5)	0.2793 (7)	0.5737 (4)	0.062 (3)	0.61 (2)
F22A	0.5122 (14)	0.2372 (4)	0.5819 (6)	0.069 (4)	0.39 (2)
F23A	0.5991 (5)	0.3159 (9)	0.5588 (6)	0.048 (3)	0.39 (2)
F24A	0.4703 (9)	0.3102 (9)	0.5044 (7)	0.036 (3)	0.39 (2)
Hf2	0.05011 (2)	0.34812 (2)	0.29307 (2)	0.02059 (4)	
O9	0.03888 (16)	0.24362 (13)	0.31851 (13)	0.0254 (5)	
O10	0.13316 (16)	0.34335 (13)	0.38817 (13)	0.0238 (5)	
C21	0.0339 (3)	0.1385 (2)	0.3698 (2)	0.0317 (9)	
F25	0.0415 (2)	0.10771 (14)	0.31083 (15)	0.0584 (8)	
F26	−0.05068 (17)	0.14064 (13)	0.37891 (17)	0.0482 (7)	
F27	0.0740 (2)	0.10128 (13)	0.42026 (15)	0.0495 (7)	
C22	0.0703 (2)	0.20906 (19)	0.36922 (19)	0.0237 (7)	
C23	0.1315 (2)	0.2302 (2)	0.42349 (19)	0.0263 (8)	
H23	0.1542	0.1991	0.4576	0.032*	
C24	0.1591 (2)	0.2953 (2)	0.42804 (18)	0.0235 (7)	
C25	0.2278 (2)	0.3170 (2)	0.48612 (19)	0.0297 (8)	
F28	0.19783 (18)	0.36545 (15)	0.52273 (14)	0.0467 (7)	
F29	0.29945 (16)	0.33921 (15)	0.46019 (13)	0.0432 (6)	
F30	0.25335 (17)	0.26747 (14)	0.52928 (12)	0.0413 (6)	
O11	−0.05268 (16)	0.33560 (13)	0.35927 (13)	0.0259 (6)	
O12	0.04500 (16)	0.44602 (13)	0.34183 (13)	0.0234 (5)	
C26	−0.1791 (2)	0.3432 (2)	0.4163 (2)	0.0296 (8)	
F31	−0.16297 (17)	0.28179 (14)	0.43867 (15)	0.0468 (7)	
F32	−0.24156 (15)	0.33971 (13)	0.36372 (14)	0.0400 (6)	
F33	−0.21122 (18)	0.37811 (15)	0.46555 (15)	0.0502 (7)	
C27	−0.0975 (2)	0.3758 (2)	0.39135 (19)	0.0248 (8)	
C28	−0.0814 (2)	0.4423 (2)	0.40295 (19)	0.0274 (8)	
H28	−0.1194	0.4677	0.4282	0.033*	
C29	−0.0091 (2)	0.4728 (2)	0.37765 (18)	0.0237 (7)	
C30	0.0080 (3)	0.5464 (2)	0.3921 (2)	0.0319 (9)	
F34	−0.0084 (3)	0.58166 (15)	0.33567 (16)	0.0698 (10)	
F35	0.09035 (17)	0.55646 (14)	0.41741 (16)	0.0496 (7)	
F36	−0.04077 (19)	0.57065 (14)	0.43865 (16)	0.0495 (7)	
O13	0.10713 (16)	0.41745 (13)	0.22774 (13)	0.0243 (5)	
O14	0.17452 (16)	0.30105 (13)	0.27081 (13)	0.0247 (5)	
C31	0.1684 (3)	0.4764 (2)	0.1407 (2)	0.0332 (9)	
F37	0.1948 (2)	0.52825 (14)	0.17918 (16)	0.0552 (8)	
F38	0.09032 (19)	0.49093 (14)	0.10948 (15)	0.0511 (7)	
F39	0.2232 (2)	0.46960 (16)	0.09337 (18)	0.0682 (10)	
C32	0.1645 (2)	0.41349 (19)	0.18522 (19)	0.0249 (7)	
C33	0.2210 (3)	0.3614 (2)	0.1775 (2)	0.0278 (8)	
H33	0.2579	0.3615	0.1413	0.033*	
C34	0.2231 (2)	0.30842 (19)	0.22395 (19)	0.0241 (7)	
C35	0.2891 (3)	0.2518 (2)	0.2196 (2)	0.0319 (9)	
F40	0.33034 (18)	0.23785 (15)	0.28025 (15)	0.0519 (7)	
F41	0.3485 (2)	0.26543 (16)	0.17714 (18)	0.0621 (9)	
F42	0.2483 (2)	0.19740 (14)	0.19608 (19)	0.0601 (9)	
O15	−0.06754 (16)	0.39771 (14)	0.24297 (13)	0.0246 (5)	
O16	0.01956 (16)	0.29709 (14)	0.19657 (13)	0.0254 (5)	
C36	−0.1771 (3)	0.4516 (2)	0.1697 (2)	0.0324 (9)	
F43	−0.25120 (16)	0.41942 (16)	0.17806 (18)	0.0560 (8)	
F44	−0.18531 (19)	0.47703 (14)	0.10659 (14)	0.0488 (7)	
F45	−0.16958 (18)	0.50112 (14)	0.21439 (14)	0.0465 (7)	
C37	−0.1003 (2)	0.40286 (19)	0.18149 (19)	0.0247 (8)	
C38	−0.0762 (2)	0.3666 (2)	0.1256 (2)	0.0268 (8)	
H38	−0.0983	0.3778	0.0797	0.032*	
C39	−0.0191 (2)	0.31374 (19)	0.13900 (19)	0.0250 (7)	
C40	−0.0013 (3)	0.2665 (2)	0.0799 (2)	0.0324 (9)	
F46	−0.0464 (3)	0.21060 (15)	0.08203 (16)	0.0623 (9)	
F47	0.0824 (2)	0.2514 (2)	0.08321 (15)	0.0695 (11)	
F48	−0.02508 (17)	0.29400 (13)	0.01846 (12)	0.0396 (6)	

### Atomic displacement parameters (Å^2^)

**Table:**

	*U*^11^	*U*^22^	*U*^33^	*U*^12^	*U*^13^	*U*^23^
Hf1	0.01424 (7)	0.02359 (8)	0.02007 (7)	0.00008 (5)	0.00223 (5)	−0.00098 (5)
O1	0.0196 (12)	0.0175 (12)	0.0244 (12)	−0.0022 (9)	0.0017 (9)	0.0004 (10)
O2	0.0238 (13)	0.0251 (13)	0.0227 (12)	−0.0015 (11)	−0.0004 (10)	0.0004 (10)
C1	0.039 (2)	0.026 (2)	0.039 (2)	0.0008 (18)	−0.0011 (18)	0.0032 (17)
F1	0.0335 (15)	0.0406 (16)	0.124 (3)	−0.0167 (13)	−0.0194 (17)	0.0279 (18)
F2	0.0397 (14)	0.0284 (12)	0.0429 (14)	0.0032 (11)	0.0039 (11)	0.0071 (10)
F3	0.134 (3)	0.0309 (15)	0.0361 (15)	0.0035 (17)	−0.0021 (18)	−0.0064 (12)
C2	0.0182 (17)	0.0268 (19)	0.0267 (18)	−0.0002 (14)	0.0070 (14)	−0.0022 (15)
C3	0.0217 (17)	0.0296 (19)	0.0239 (18)	0.0024 (15)	0.0035 (14)	0.0013 (15)
C4	0.0158 (16)	0.0309 (19)	0.0223 (17)	0.0007 (14)	0.0045 (13)	−0.0038 (14)
C5	0.0246 (19)	0.035 (2)	0.0245 (18)	−0.0006 (16)	−0.0007 (14)	−0.0025 (16)
F4	0.0577 (18)	0.0619 (18)	0.0303 (13)	−0.0183 (15)	−0.0151 (12)	0.0127 (13)
F5	0.0524 (17)	0.076 (2)	0.0462 (16)	0.0190 (16)	−0.0125 (13)	−0.0371 (15)
F6	0.0255 (12)	0.0609 (17)	0.0379 (14)	−0.0113 (12)	−0.0028 (10)	−0.0009 (12)
O3	0.0188 (12)	0.0264 (13)	0.0255 (13)	−0.0040 (10)	0.0062 (10)	−0.0030 (10)
O4	0.0182 (12)	0.0254 (13)	0.0258 (13)	−0.0026 (10)	0.0024 (10)	−0.0003 (10)
C6	0.026 (2)	0.032 (2)	0.038 (2)	0.0018 (17)	0.0136 (16)	0.0044 (17)
F7	0.0374 (14)	0.0361 (14)	0.0643 (18)	0.0058 (11)	0.0229 (13)	0.0221 (13)
F8	0.0199 (12)	0.0537 (16)	0.0552 (16)	−0.0074 (11)	0.0060 (11)	0.0047 (13)
F9	0.0392 (14)	0.0495 (16)	0.0483 (15)	0.0039 (12)	0.0266 (12)	0.0013 (13)
C7	0.0168 (16)	0.0292 (19)	0.0229 (17)	0.0027 (14)	0.0029 (13)	0.0045 (14)
C8	0.0261 (19)	0.026 (2)	0.040 (2)	0.0032 (16)	0.0128 (16)	−0.0035 (17)
C9	0.0198 (17)	0.0252 (18)	0.0276 (18)	0.0020 (14)	0.0000 (14)	−0.0015 (15)
C10	0.035 (2)	0.025 (2)	0.042 (2)	−0.0024 (17)	0.0084 (18)	−0.0042 (17)
F10	0.119 (7)	0.047 (4)	0.049 (4)	−0.022 (5)	−0.040 (4)	−0.001 (3)
F11	0.035 (2)	0.021 (2)	0.067 (4)	0.0040 (18)	0.006 (2)	0.005 (2)
F12	0.051 (4)	0.029 (3)	0.111 (6)	−0.014 (2)	0.040 (4)	−0.018 (3)
F10A	0.131 (10)	0.053 (6)	0.058 (7)	−0.007 (7)	−0.018 (7)	0.020 (5)
F11A	0.046 (6)	0.048 (6)	0.111 (9)	−0.019 (4)	0.036 (6)	−0.035 (6)
F12A	0.053 (6)	0.038 (5)	0.040 (5)	−0.017 (5)	0.027 (5)	−0.015 (4)
O5	0.0195 (12)	0.0276 (14)	0.0260 (13)	0.0024 (10)	0.0074 (10)	0.0027 (10)
O6	0.0181 (12)	0.0296 (14)	0.0301 (14)	0.0014 (11)	0.0043 (10)	0.0012 (11)
C11	0.033 (2)	0.034 (2)	0.033 (2)	0.0026 (17)	0.0123 (17)	0.0064 (17)
F13	0.0559 (17)	0.0321 (14)	0.0493 (16)	−0.0095 (12)	0.0058 (13)	0.0042 (12)
F14	0.0388 (14)	0.0513 (17)	0.0469 (15)	0.0044 (13)	−0.0019 (12)	0.0151 (13)
F15	0.0627 (19)	0.0538 (18)	0.0585 (18)	0.0189 (15)	0.0395 (15)	0.0211 (14)
C12	0.0247 (18)	0.031 (2)	0.0259 (18)	0.0001 (16)	0.0044 (14)	−0.0017 (15)
C13	0.025 (2)	0.043 (2)	0.039 (2)	0.0070 (18)	0.0166 (17)	0.0074 (19)
C14	0.0182 (17)	0.034 (2)	0.038 (2)	0.0053 (16)	0.0047 (15)	0.0012 (17)
C15	0.040 (3)	0.047 (3)	0.068 (4)	0.021 (2)	0.024 (2)	0.020 (3)
F16	0.067 (6)	0.072 (6)	0.095 (6)	0.024 (4)	0.039 (5)	0.045 (5)
F17	0.014 (2)	0.043 (3)	0.051 (3)	0.009 (2)	0.005 (2)	0.003 (2)
F18	0.055 (4)	0.066 (5)	0.100 (6)	0.029 (4)	−0.015 (4)	−0.046 (4)
F16A	0.118 (8)	0.122 (8)	0.178 (9)	0.055 (6)	0.093 (7)	0.052 (7)
F17A	0.100 (6)	0.061 (5)	0.122 (7)	0.049 (5)	−0.008 (5)	−0.029 (5)
F18A	0.055 (5)	0.075 (7)	0.104 (6)	0.030 (4)	−0.038 (4)	0.009 (5)
O7	0.0180 (12)	0.0305 (14)	0.0217 (12)	0.0018 (10)	0.0019 (9)	−0.0019 (10)
O8	0.0231 (13)	0.0285 (14)	0.0213 (12)	0.0051 (11)	0.0009 (10)	−0.0021 (10)
C16	0.0260 (19)	0.028 (2)	0.035 (2)	0.0030 (16)	−0.0015 (16)	−0.0009 (16)
F19	0.0212 (12)	0.0384 (14)	0.077 (2)	0.0039 (11)	0.0022 (12)	−0.0007 (13)
F20	0.0564 (17)	0.0603 (18)	0.0382 (14)	0.0324 (15)	−0.0023 (12)	0.0073 (13)
F21	0.0462 (15)	0.0266 (13)	0.0557 (16)	0.0102 (11)	0.0005 (12)	−0.0081 (11)
C17	0.0169 (16)	0.0250 (19)	0.0309 (19)	−0.0006 (14)	0.0029 (14)	0.0003 (15)
C18	0.0228 (18)	0.030 (2)	0.0247 (18)	0.0027 (15)	−0.0022 (14)	−0.0015 (15)
C19	0.0219 (17)	0.0248 (18)	0.0259 (18)	−0.0003 (15)	0.0029 (14)	−0.0005 (15)
C20	0.034 (2)	0.039 (2)	0.026 (2)	0.0125 (19)	−0.0004 (16)	−0.0071 (17)
F22	0.050 (5)	0.054 (5)	0.028 (3)	0.009 (4)	0.001 (3)	−0.008 (3)
F23	0.067 (5)	0.032 (3)	0.053 (3)	0.002 (3)	0.009 (3)	−0.015 (2)
F24	0.039 (3)	0.093 (6)	0.050 (3)	0.040 (4)	−0.011 (2)	−0.036 (4)
F22A	0.108 (10)	0.039 (5)	0.062 (6)	0.020 (6)	0.014 (6)	−0.001 (4)
F23A	0.029 (4)	0.072 (7)	0.043 (5)	0.007 (4)	0.004 (3)	−0.035 (5)
F24A	0.026 (5)	0.052 (7)	0.027 (4)	0.005 (4)	−0.011 (3)	−0.019 (4)
Hf2	0.01420 (7)	0.02665 (8)	0.02060 (7)	−0.00038 (6)	0.00031 (5)	−0.00107 (6)
O9	0.0186 (12)	0.0308 (14)	0.0260 (13)	0.0009 (11)	−0.0007 (10)	0.0004 (11)
O10	0.0186 (12)	0.0286 (14)	0.0234 (12)	−0.0018 (10)	−0.0016 (9)	0.0003 (11)
C21	0.037 (2)	0.0241 (19)	0.034 (2)	0.0031 (17)	0.0047 (17)	0.0010 (16)
F25	0.098 (3)	0.0342 (15)	0.0448 (16)	−0.0029 (15)	0.0155 (16)	−0.0124 (12)
F26	0.0317 (14)	0.0352 (14)	0.078 (2)	−0.0096 (11)	0.0088 (13)	0.0022 (13)
F27	0.0558 (17)	0.0322 (14)	0.0580 (17)	−0.0022 (12)	−0.0043 (14)	0.0149 (12)
C22	0.0181 (16)	0.0286 (19)	0.0253 (18)	0.0033 (14)	0.0073 (13)	−0.0009 (14)
C23	0.0244 (18)	0.034 (2)	0.0212 (17)	0.0028 (16)	0.0037 (14)	0.0032 (15)
C24	0.0149 (16)	0.037 (2)	0.0192 (16)	0.0037 (15)	0.0051 (13)	−0.0022 (15)
C25	0.0216 (18)	0.044 (2)	0.0229 (18)	0.0022 (17)	−0.0005 (14)	−0.0011 (17)
F28	0.0448 (15)	0.0562 (17)	0.0366 (14)	0.0125 (13)	−0.0071 (11)	−0.0217 (12)
F29	0.0226 (12)	0.0686 (19)	0.0369 (14)	−0.0117 (12)	−0.0035 (10)	0.0035 (13)
F30	0.0379 (14)	0.0540 (16)	0.0293 (12)	0.0035 (12)	−0.0089 (10)	0.0052 (11)
O11	0.0196 (12)	0.0306 (14)	0.0277 (13)	−0.0020 (11)	0.0033 (10)	−0.0004 (11)
O12	0.0176 (12)	0.0299 (14)	0.0227 (12)	−0.0016 (10)	0.0020 (9)	−0.0018 (10)
C26	0.0196 (18)	0.035 (2)	0.036 (2)	0.0030 (16)	0.0073 (15)	0.0095 (17)
F31	0.0313 (13)	0.0470 (16)	0.0641 (18)	0.0078 (12)	0.0139 (12)	0.0304 (14)
F32	0.0199 (11)	0.0465 (15)	0.0528 (16)	−0.0037 (10)	−0.0006 (10)	0.0140 (12)
F33	0.0407 (15)	0.0632 (19)	0.0515 (16)	−0.0037 (14)	0.0281 (13)	−0.0019 (14)
C27	0.0170 (16)	0.035 (2)	0.0225 (17)	0.0024 (15)	0.0005 (13)	0.0054 (15)
C28	0.0211 (18)	0.037 (2)	0.0247 (18)	0.0028 (16)	0.0041 (14)	0.0000 (16)
C29	0.0192 (17)	0.033 (2)	0.0178 (16)	0.0016 (15)	−0.0015 (13)	−0.0017 (14)
C30	0.030 (2)	0.036 (2)	0.030 (2)	−0.0020 (17)	0.0048 (16)	−0.0046 (17)
F34	0.128 (3)	0.0372 (16)	0.0409 (16)	−0.0097 (18)	−0.0050 (18)	0.0070 (13)
F35	0.0303 (13)	0.0480 (16)	0.0709 (19)	−0.0102 (12)	0.0076 (13)	−0.0248 (14)
F36	0.0466 (16)	0.0405 (15)	0.0652 (18)	−0.0007 (13)	0.0237 (14)	−0.0183 (13)
O13	0.0201 (12)	0.0271 (14)	0.0263 (13)	0.0027 (11)	0.0049 (10)	0.0000 (11)
O14	0.0171 (12)	0.0301 (14)	0.0267 (13)	0.0022 (10)	0.0014 (10)	0.0026 (11)
C31	0.032 (2)	0.032 (2)	0.037 (2)	0.0057 (17)	0.0126 (17)	0.0044 (17)
F37	0.073 (2)	0.0308 (14)	0.0606 (18)	−0.0165 (14)	0.0039 (15)	0.0036 (13)
F38	0.0538 (17)	0.0460 (16)	0.0515 (17)	0.0079 (14)	−0.0031 (13)	0.0202 (13)
F39	0.088 (2)	0.0551 (19)	0.072 (2)	0.0272 (17)	0.0549 (19)	0.0304 (16)
C32	0.0234 (18)	0.0268 (19)	0.0244 (18)	−0.0001 (15)	0.0019 (14)	0.0023 (15)
C33	0.0253 (19)	0.029 (2)	0.031 (2)	0.0011 (16)	0.0083 (15)	0.0017 (16)
C34	0.0163 (16)	0.0255 (18)	0.0297 (19)	−0.0008 (14)	−0.0008 (14)	−0.0018 (15)
C35	0.0214 (19)	0.029 (2)	0.045 (2)	0.0055 (16)	0.0050 (17)	0.0053 (18)
F40	0.0387 (15)	0.0586 (18)	0.0557 (17)	0.0214 (13)	−0.0067 (13)	0.0100 (14)
F41	0.0525 (18)	0.0535 (18)	0.088 (2)	0.0253 (15)	0.0425 (17)	0.0226 (17)
F42	0.0473 (17)	0.0362 (15)	0.095 (2)	0.0051 (13)	−0.0006 (16)	−0.0217 (16)
O15	0.0162 (12)	0.0344 (15)	0.0227 (12)	0.0007 (10)	−0.0005 (10)	−0.0025 (11)
O16	0.0210 (12)	0.0320 (14)	0.0223 (13)	0.0014 (11)	−0.0022 (10)	−0.0019 (11)
C36	0.026 (2)	0.034 (2)	0.035 (2)	0.0081 (17)	−0.0081 (16)	−0.0049 (17)
F43	0.0200 (12)	0.0560 (18)	0.091 (2)	0.0033 (12)	−0.0011 (13)	0.0019 (16)
F44	0.0544 (17)	0.0492 (16)	0.0402 (15)	0.0228 (14)	−0.0073 (12)	0.0013 (12)
F45	0.0479 (16)	0.0421 (15)	0.0472 (15)	0.0167 (13)	−0.0056 (12)	−0.0134 (12)
C37	0.0177 (17)	0.0291 (19)	0.0261 (18)	−0.0006 (14)	−0.0039 (14)	0.0001 (15)
C38	0.0203 (17)	0.035 (2)	0.0239 (18)	0.0001 (15)	−0.0025 (14)	0.0000 (15)
C39	0.0190 (17)	0.0292 (19)	0.0268 (18)	−0.0045 (15)	0.0024 (14)	−0.0021 (15)
C40	0.029 (2)	0.041 (2)	0.0262 (19)	0.0044 (18)	−0.0003 (15)	−0.0060 (17)
F46	0.104 (3)	0.0360 (16)	0.0479 (17)	−0.0099 (17)	0.0146 (17)	−0.0098 (13)
F47	0.0433 (16)	0.121 (3)	0.0426 (16)	0.0370 (18)	−0.0044 (13)	−0.0322 (18)
F48	0.0481 (15)	0.0465 (15)	0.0239 (12)	0.0037 (12)	0.0024 (10)	−0.0037 (10)

### Geometric parameters (Å, º)

**Table:**

Hf1—O1	2.169 (2)	C20—F24	1.269 (7)
Hf1—O2	2.143 (2)	C20—F22A	1.292 (7)
Hf1—O3	2.156 (2)	C20—F23A	1.390 (7)
Hf1—O4	2.179 (3)	C20—F24A	1.336 (8)
Hf1—O5	2.140 (2)	Hf2—O9	2.166 (3)
Hf1—O6	2.202 (3)	Hf2—O10	2.134 (2)
Hf1—O7	2.180 (2)	Hf2—O11	2.154 (3)
Hf1—O8	2.153 (2)	Hf2—O12	2.188 (3)
O1—C2	1.259 (4)	Hf2—O13	2.135 (3)
O2—C4	1.267 (5)	Hf2—O14	2.210 (2)
C1—F1	1.325 (5)	Hf2—O15	2.193 (2)
C1—F2	1.327 (5)	Hf2—O16	2.149 (3)
C1—F3	1.331 (5)	O9—C22	1.260 (4)
C1—C2	1.526 (6)	O10—C24	1.275 (5)
C2—C3	1.394 (5)	C21—F25	1.321 (5)
C3—H3	0.9500	C21—F26	1.325 (5)
C3—C4	1.385 (5)	C21—F27	1.331 (5)
C4—C5	1.530 (5)	C21—C22	1.523 (6)
C5—F4	1.326 (5)	C22—C23	1.402 (5)
C5—F5	1.320 (5)	C23—H23	0.9500
C5—F6	1.321 (5)	C23—C24	1.373 (6)
O3—C7	1.258 (4)	C24—C25	1.524 (5)
O4—C9	1.255 (4)	C25—F28	1.317 (5)
C6—F7	1.327 (5)	C25—F29	1.332 (5)
C6—F8	1.327 (5)	C25—F30	1.334 (5)
C6—F9	1.325 (5)	O11—C27	1.267 (5)
C6—C7	1.530 (5)	O12—C29	1.259 (4)
C7—C8	1.374 (6)	C26—F31	1.322 (5)
C8—H8	0.9500	C26—F32	1.326 (5)
C8—C9	1.393 (5)	C26—F33	1.325 (5)
C9—C10	1.531 (5)	C26—C27	1.535 (5)
C10—F10	1.254 (9)	C27—C28	1.372 (6)
C10—F11	1.351 (7)	C28—H28	0.9500
C10—F12	1.314 (7)	C28—C29	1.399 (5)
C10—F10A	1.284 (8)	C29—C30	1.522 (6)
C10—F11A	1.355 (8)	C30—F34	1.309 (5)
C10—F12A	1.367 (8)	C30—F35	1.319 (5)
O5—C12	1.273 (4)	C30—F36	1.328 (5)
O6—C14	1.247 (5)	O13—C32	1.273 (4)
C11—F13	1.323 (5)	O14—C34	1.247 (4)
C11—F14	1.316 (5)	C31—F37	1.320 (5)
C11—F15	1.328 (5)	C31—F38	1.315 (5)
C11—C12	1.535 (6)	C31—F39	1.319 (5)
C12—C13	1.377 (6)	C31—C32	1.536 (5)
C13—H13	0.9500	C32—C33	1.375 (5)
C13—C14	1.391 (6)	C33—H33	0.9500
C14—C15	1.533 (6)	C33—C34	1.394 (5)
C15—F16	1.252 (10)	C34—C35	1.528 (5)
C15—F17	1.386 (8)	C35—F40	1.309 (5)
C15—F18	1.301 (9)	C35—F41	1.323 (5)
C15—F16A	1.288 (7)	C35—F42	1.316 (5)
C15—F17A	1.354 (7)	O15—C37	1.253 (4)
C15—F18A	1.338 (7)	O16—C39	1.256 (4)
O7—C17	1.253 (4)	C36—F43	1.330 (5)
O8—C19	1.256 (4)	C36—F44	1.326 (5)
C16—F19	1.335 (5)	C36—F45	1.319 (5)
C16—F20	1.327 (5)	C36—C37	1.527 (5)
C16—F21	1.312 (5)	C37—C38	1.393 (5)
C16—C17	1.536 (5)	C38—H38	0.9500
C17—C18	1.390 (5)	C38—C39	1.381 (5)
C18—H18	0.9500	C39—C40	1.539 (5)
C18—C19	1.382 (5)	C40—F46	1.321 (5)
C19—C20	1.517 (5)	C40—F47	1.312 (5)
C20—F22	1.301 (9)	C40—F48	1.332 (5)
C20—F23	1.396 (8)		
			
O1—Hf1—O4	141.51 (9)	F24—C20—C19	115.7 (4)
O1—Hf1—O6	74.26 (9)	F24—C20—F22	112.2 (7)
O1—Hf1—O7	115.03 (9)	F24—C20—F23	104.3 (6)
O2—Hf1—O1	79.10 (9)	F22A—C20—C19	115.2 (6)
O2—Hf1—O3	80.29 (10)	F22A—C20—F23A	106.0 (6)
O2—Hf1—O4	73.14 (10)	F22A—C20—F24A	110.0 (7)
O2—Hf1—O6	72.25 (10)	F23A—C20—C19	104.9 (5)
O2—Hf1—O7	142.70 (9)	F24A—C20—C19	116.0 (9)
O2—Hf1—O8	141.22 (10)	F24A—C20—F23A	103.3 (7)
O3—Hf1—O1	71.72 (9)	O9—Hf2—O12	139.70 (10)
O3—Hf1—O4	77.68 (9)	O9—Hf2—O14	73.87 (10)
O3—Hf1—O6	139.51 (10)	O9—Hf2—O15	117.38 (10)
O3—Hf1—O7	72.90 (10)	O10—Hf2—O9	79.18 (10)
O4—Hf1—O6	119.96 (10)	O10—Hf2—O11	83.08 (10)
O4—Hf1—O7	76.13 (9)	O10—Hf2—O12	73.06 (10)
O5—Hf1—O1	143.59 (9)	O10—Hf2—O13	107.70 (10)
O5—Hf1—O2	109.99 (10)	O10—Hf2—O14	72.91 (10)
O5—Hf1—O3	143.43 (10)	O10—Hf2—O15	142.29 (10)
O5—Hf1—O4	72.53 (9)	O10—Hf2—O16	142.40 (10)
O5—Hf1—O6	75.39 (10)	O11—Hf2—O9	70.66 (10)
O5—Hf1—O7	79.69 (9)	O11—Hf2—O12	77.41 (10)
O5—Hf1—O8	80.44 (10)	O11—Hf2—O14	140.16 (10)
O7—Hf1—O6	143.35 (9)	O11—Hf2—O15	72.67 (10)
O8—Hf1—O1	72.62 (10)	O12—Hf2—O14	122.75 (9)
O8—Hf1—O3	114.06 (10)	O12—Hf2—O15	73.74 (9)
O8—Hf1—O4	143.20 (10)	O13—Hf2—O9	144.60 (10)
O8—Hf1—O6	74.88 (10)	O13—Hf2—O11	143.61 (10)
O8—Hf1—O7	74.82 (9)	O13—Hf2—O12	73.12 (9)
C2—O1—Hf1	132.6 (2)	O13—Hf2—O14	75.27 (9)
C4—O2—Hf1	132.7 (2)	O13—Hf2—O15	78.88 (9)
F1—C1—F2	107.3 (4)	O13—Hf2—O16	81.55 (10)
F1—C1—F3	109.1 (4)	O15—Hf2—O14	142.20 (9)
F1—C1—C2	110.2 (3)	O16—Hf2—O9	73.96 (10)
F2—C1—F3	106.3 (4)	O16—Hf2—O11	111.38 (10)
F2—C1—C2	113.5 (4)	O16—Hf2—O12	142.55 (10)
F3—C1—C2	110.3 (4)	O16—Hf2—O14	74.66 (9)
O1—C2—C1	112.8 (3)	O16—Hf2—O15	74.65 (10)
O1—C2—C3	127.6 (4)	C22—O9—Hf2	132.8 (2)
C3—C2—C1	119.6 (3)	C24—O10—Hf2	132.9 (2)
C2—C3—H3	120.1	F25—C21—F26	108.1 (4)
C4—C3—C2	119.8 (4)	F25—C21—F27	107.7 (3)
C4—C3—H3	120.1	F25—C21—C22	111.2 (3)
O2—C4—C3	128.1 (3)	F26—C21—F27	107.4 (3)
O2—C4—C5	112.4 (3)	F26—C21—C22	109.5 (3)
C3—C4—C5	119.4 (3)	F27—C21—C22	112.7 (3)
F4—C5—C4	113.2 (3)	O9—C22—C21	114.1 (3)
F5—C5—C4	110.5 (3)	O9—C22—C23	126.4 (4)
F5—C5—F4	108.4 (3)	C23—C22—C21	119.5 (3)
F5—C5—F6	106.7 (4)	C22—C23—H23	119.6
F6—C5—C4	110.8 (3)	C24—C23—C22	120.9 (4)
F6—C5—F4	106.9 (3)	C24—C23—H23	119.6
C7—O3—Hf1	134.4 (2)	O10—C24—C23	127.3 (3)
C9—O4—Hf1	133.2 (2)	O10—C24—C25	112.4 (3)
F7—C6—F8	108.1 (4)	C23—C24—C25	120.2 (3)
F7—C6—C7	110.7 (3)	F28—C25—C24	111.2 (3)
F8—C6—C7	109.5 (3)	F28—C25—F29	107.9 (4)
F9—C6—F7	107.7 (3)	F28—C25—F30	107.7 (3)
F9—C6—F8	107.5 (3)	F29—C25—C24	110.1 (3)
F9—C6—C7	113.1 (3)	F29—C25—F30	106.9 (3)
O3—C7—C6	113.3 (3)	F30—C25—C24	112.8 (3)
O3—C7—C8	127.0 (3)	C27—O11—Hf2	133.7 (2)
C8—C7—C6	119.7 (3)	C29—O12—Hf2	132.8 (2)
C7—C8—H8	119.9	F31—C26—F32	107.4 (4)
C7—C8—C9	120.3 (4)	F31—C26—F33	108.9 (3)
C9—C8—H8	119.9	F31—C26—C27	111.9 (3)
O4—C9—C8	127.3 (4)	F32—C26—C27	108.7 (3)
O4—C9—C10	115.5 (3)	F33—C26—F32	107.2 (3)
C8—C9—C10	117.2 (3)	F33—C26—C27	112.5 (3)
F10—C10—C9	112.7 (6)	O11—C27—C26	112.8 (3)
F10—C10—F11	107.8 (7)	O11—C27—C28	127.2 (3)
F10—C10—F12	113.1 (6)	C28—C27—C26	120.0 (3)
F11—C10—C9	108.5 (4)	C27—C28—H28	119.9
F12—C10—C9	112.0 (4)	C27—C28—C29	120.1 (4)
F12—C10—F11	102.0 (5)	C29—C28—H28	119.9
F10A—C10—C9	115.0 (8)	O12—C29—C28	127.0 (4)
F10A—C10—F11A	110.2 (7)	O12—C29—C30	113.9 (3)
F10A—C10—F12A	107.8 (8)	C28—C29—C30	119.1 (3)
F11A—C10—C9	111.0 (6)	F34—C30—C29	110.9 (3)
F11A—C10—F12A	101.1 (6)	F34—C30—F35	108.7 (4)
F12A—C10—C9	110.8 (7)	F34—C30—F36	107.8 (4)
C12—O5—Hf1	134.6 (3)	F35—C30—C29	110.9 (3)
C14—O6—Hf1	133.7 (3)	F35—C30—F36	105.8 (3)
F13—C11—F15	107.8 (4)	F36—C30—C29	112.6 (3)
F13—C11—C12	110.4 (3)	C32—O13—Hf2	134.6 (2)
F14—C11—F13	107.6 (4)	C34—O14—Hf2	133.4 (2)
F14—C11—F15	107.9 (4)	F37—C31—C32	110.8 (3)
F14—C11—C12	111.2 (3)	F38—C31—F37	107.1 (4)
F15—C11—C12	111.8 (3)	F38—C31—F39	108.2 (4)
O5—C12—C11	112.7 (3)	F38—C31—C32	110.8 (3)
O5—C12—C13	126.9 (4)	F39—C31—F37	107.5 (4)
C13—C12—C11	120.3 (3)	F39—C31—C32	112.1 (3)
C12—C13—H13	120.6	O13—C32—C31	112.8 (3)
C12—C13—C14	118.9 (4)	O13—C32—C33	127.2 (4)
C14—C13—H13	120.6	C33—C32—C31	120.0 (3)
O6—C14—C13	126.3 (4)	C32—C33—H33	120.6
O6—C14—C15	114.3 (4)	C32—C33—C34	118.7 (3)
C13—C14—C15	119.4 (4)	C34—C33—H33	120.6
F16—C15—C14	116.5 (6)	O14—C34—C33	126.3 (3)
F16—C15—F17	105.5 (7)	O14—C34—C35	113.7 (3)
F16—C15—F18	112.7 (8)	C33—C34—C35	120.1 (3)
F17—C15—C14	108.2 (4)	F40—C35—C34	111.5 (4)
F18—C15—C14	110.1 (5)	F40—C35—F41	108.2 (3)
F18—C15—F17	102.7 (6)	F40—C35—F42	107.6 (4)
F16A—C15—C14	114.5 (6)	F41—C35—C34	112.5 (3)
F16A—C15—F17A	109.4 (7)	F42—C35—C34	110.2 (3)
F16A—C15—F18A	111.0 (7)	F42—C35—F41	106.6 (4)
F17A—C15—C14	108.3 (5)	C37—O15—Hf2	133.5 (2)
F18A—C15—C14	109.6 (6)	C39—O16—Hf2	133.8 (3)
F18A—C15—F17A	103.4 (6)	F43—C36—C37	108.9 (3)
C17—O7—Hf1	134.0 (2)	F44—C36—F43	107.7 (3)
C19—O8—Hf1	134.9 (2)	F44—C36—C37	112.4 (3)
F19—C16—C17	109.3 (3)	F45—C36—F43	107.5 (4)
F20—C16—F19	107.5 (3)	F45—C36—F44	108.3 (4)
F20—C16—C17	112.0 (3)	F45—C36—C37	111.9 (3)
F21—C16—F19	107.3 (3)	O15—C37—C36	114.8 (3)
F21—C16—F20	108.5 (3)	O15—C37—C38	126.4 (3)
F21—C16—C17	112.0 (3)	C38—C37—C36	118.8 (3)
O7—C17—C16	114.4 (3)	C37—C38—H38	121.0
O7—C17—C18	126.9 (3)	C39—C38—C37	118.0 (3)
C18—C17—C16	118.6 (3)	C39—C38—H38	121.0
C17—C18—H18	121.0	O16—C39—C38	126.9 (4)
C19—C18—C17	118.1 (3)	O16—C39—C40	113.7 (3)
C19—C18—H18	121.0	C38—C39—C40	119.3 (3)
O8—C19—C18	126.4 (3)	F46—C40—C39	111.4 (3)
O8—C19—C20	113.8 (3)	F46—C40—F48	106.7 (3)
C18—C19—C20	119.7 (3)	F47—C40—C39	110.8 (3)
F22—C20—C19	112.0 (7)	F47—C40—F46	108.2 (4)
F22—C20—F23	104.1 (6)	F47—C40—F48	108.1 (4)
F23—C20—C19	107.6 (4)	F48—C40—C39	111.4 (3)

### Hydrogen-bond geometry (Å, º)

**Table:**

*D*—H···*A*	*D*—H	H···*A*	*D*···*A*	*D*—H···*A*
C3—H3···F23*A*^i^	0.95	2.57	3.374 (10)	143
C8—H8···F5^ii^	0.95	2.50	3.427 (5)	166
C33—H33···F11*A*^iii^	0.95	2.49	3.292 (9)	142
C13—H13···F35^iii^	0.95	2.65	3.528 (5)	154
C3—H3···F24^i^	0.95	2.68	3.375 (8)	130

Symmetry codes: (i) *x*, −*y*+1/2, *z*+1/2; (ii) −*x*+1, −*y*+1, −*z*+2; (iii) −*x*+1, −*y*+1, −*z*+1.
